# TMEM206 Contributes to Cancer Hallmark Functions in Colorectal Cancer Cells and Is Regulated by p53 in a p21-Dependent Manner

**DOI:** 10.3390/cells13221825

**Published:** 2024-11-05

**Authors:** Korollus Melek, Barbara Hauert, Sven Kappel

**Affiliations:** Institute of Biochemistry and Molecular Medicine, University of Bern, 3012 Bern, Switzerland; korollus.melek@unibe.ch (K.M.); barbara.hauert@unibe.ch (B.H.)

**Keywords:** TMEM206, ion channel, colorectal cancer, p53, p21, acid-induced cell death

## Abstract

Acid-induced ion flux plays a role in pathologies where tissue acidification is prevalent, including cancer. In 2019, TMEM206 was identified as the molecular component of acid-induced chloride flux. Localizing to the plasma membrane, TMEM206 contributes to cellular processes like acid-induced cell death. Since over 50% of human cancers carry loss of function mutations in the p53 gene, we aimed to analyze how TMEM206 is regulated by p53 and its role in cancer hallmark function and acid-induced cell death in HCT116 colorectal cancer (CRC) cells. We generated p53-deficient HCT116 cells and assessed TMEM206-mediated Cl^−^ currents and transcriptional regulation using the patch-clamp and a dual-luciferase reporter assay, respectively. To investigate the contribution of TMEM206 to cancer hallmark functions, we performed migration and metabolic activity assays. The role of TMEM206 in p53-mediated acid-induced cell death was assessed with cell death assays. The TMEM206 mRNA level was significantly elevated in human primary CRC tumors. TMEM206 knockout increased acid-induced cell death and reduced proliferation and migration, indicating a role for TMEM206 in these cancer hallmark functions. Furthermore, we observed increased TMEM206 mRNA levels and currents in HCT116 p53 knockout cells. This phenotype can be rescued by transient overexpression of p53 but not by overexpression of dysfunctional p53. In addition, our data suggest that TMEM206 may mediate cancer hallmark functions within p53-associated pathways. TMEM206 promoter activity is not altered by p53 overexpression. Conversely, knockout of p21, a major target gene of p53, increased TMEM206-mediated currents, suggesting expression control of TMEM206 by p21 downstream signaling. Our results show that in colorectal cancer cells, TMEM206 expression is elevated, contributes to cancer hallmark functions, and its regulation is dependent on p53 through a p21-dependent mechanism.

## 1. Introduction

The tumor microenvironment is characterized by a heterogeneous cellular composition and by low pH once the tumor has exceeded a certain volume [1]. Changes in cancer cell metabolism towards glycolysis, the so-called Warburg effect, acidifies the tumor surrounding by increased lactate production [2]. Low extracellular pH drives changes in cellular signaling, leading to adaption to acidosis, which gives cancer cells a selection advantage [3]. In this context, ion channels come into play. Being expressed at the plasma membrane level, ion channels transduce extrinsic signals into the cell [4]. On top of that, many ion channels are dysregulated in cancer and contribute to cancer hallmark functions [5]. Certain types of ion channels are sensitive to changes in extracellular pH. Besides acid-sensing ion channels (ASICs), so-called acid-sensitive outwardly rectifying anion (ASOR) channels [6] or proton-activated outwardly rectifying anion (PAORAC) channels [7] are activated by low pH and mediate chloride (Cl^−^) flux. Cl^−^ is the most abundant anion in the human body and regulates physiological functions, i.e., cell volume, vesicular acidification, transepithelial transport, and cellular signaling [8,9]. In 2019, the molecular component of ASOR/PAORAC was identified as TMEM206 [10,11].

Since the molecular identification of TMEM206, its role in physiological functions and human pathologies has been under intense investigation. TMEM206 has been shown to play a role in acid-induced cell death in human cells [6,10,12]. In primary rat cortical neurons, knockout of TMEM206 attenuates acid-induced cell death and brain damage in mice after ischemic stroke and reduces neuronal death upon acid treatment [13]. Additionally, TMEM206 prevents endosomal hyper-acidification [14] and regulates macropinosome shrinkage and acidification [15].

Recent research demonstrates that TMEM206 expression is increased in human primary colorectal tumors and exclusively conducts acid-induced Cl^−^ current in colorectal cancer (CRC) cells [16,17]. In hepatocellular carcinoma, high TMEM206 expression correlates with poor overall survival [18,19]. In human osteosarcoma cells, downregulation of TMEM206 inhibits malignant properties, i.e., proliferation, migration, and invasion [20].

In contrast to its contribution to cellular functions, not much is known about the regulatory mechanisms of TMEM206 in cancer. Zhang et al. report that TMEM206 may be regulated by micro-RNAs (miR) I-miR-325 and I-miR-510-5p, as high expression of these miRs was favorable for overall survival [18].

In cancer, p53 is frequently mutated, and 80% of these mutations are within a few hotspot amino acids that hinder p53’s ability to bind to DNA [21]. Dysfunctional p53 is linked to altered gene expression in cancer and contributes to several cancer hallmarks, including increased proliferation and migration and resistance to cell death [22]. In contrast to its function as a transcription factor, p53 was also reported to interact with other proteins that are crucial for apoptosis or necrosis [23].

One of the major target genes of p53 is the cyclin-dependent kinase inhibitor 1 (CDKN1A), also known as p21Cip1/Waf1 [24,25], which in turn alters cell cycle progression via the DREAM pathway [26,27,28]. In addition, several studies link the loss of p53 function in cancer to changes in ion channel expression [29,30,31].

In our study, we investigated the role of TMEM206 in cancer hallmark functions and the regulation of TMEM206 by p53 as well as the underlying mechanism in CRC cells.

## 2. Materials and Methods

### 2.1. Cell Culture

HCT116 cells (human colorectal cancer cell line) were purchased from the American Type Culture Collection (ATCC, Manassas, VA, USA), and cells were cultivated in McCoy’s 5A medium (Thermo Fisher Scientific, Waltham, MA, USA) supplemented with 10% fetal bovine serum (FBS; Sigma, St. Louis, MO, USA) and passaged every two to three days. Prostate cancer LNCaP cells (ATCC) were cultivated in RPMI 1640 medium (Thermo Fisher Scientific, Waltham, MA, USA) supplemented with 10% FBS and passaged twice per week. All cells were incubated at 37 °C and 5% CO_2_ in a humidified environment.

### 2.2. Transfection

If not stated otherwise, cells were transfected with the 4D-Nucleofector X-unit (Lonza, Basel, Switzerland) and the SE cell line Nucleofector (Lonza, V4XC-1024) kit for HCT116 cells or the SF cell line Nucleofector (Lonza, V4XC-2024) kit for LNCaP cells according to the manufacturer’s instructions.

### 2.3. CRISPR Cas9 KO

The HCT116 TMEM206 knockout (TMEM206 KO) clones were already used in a previous study by our group [17]. HCT116 p53 knockout (p53 KO) and HCT116 p53/TMEM206 double knockout (DKO) cells were generated with the CRISPR/Cas9 system. Two guide RNAs (gRNAs) targeting the human p53 coding region were designed to delete around 355bp between exon 1 and exon 3. For the knockout of TMEM206, 67 to 68 base pairs were deleted in exon 1 (Chr 1: 212’414’732 to 212’414’798 or 212’414’731 to 212’414’798) using two gRNAs. These clones had already been used in a previous study from our group [17]. gRNA design was performed using CRISPOR [32] and E-CRISP [33]. Corresponding gRNA sense and antisense oligos (Table 1) were cloned into pSpCas9(BB)-2A-GFP (Addgene, Watertown, MA, USA) according to the protocol available on Addgene [34]. HCT116 TMEM206 knockout clones were subsequently generated from the HCT116 p53 knockout clone 2, resulting in the establishment of two HCT116 p53/TMEM206 double knockout cell lines (DKO1 and DKO2).

First, 2 × 10^6^ cells were transfected with 2 µg of two gRNA plasmids corresponding to the target gene. Then, 24 h later, the cells were sorted in the ASTRIOS flow cytometry sorter (Beckman Coulter, Brea, CA, USA) for GFP. Single cells were seeded in two 96-well plates, and the rest of the sorted cells were collected and seeded in 10 cm dishes in different dilutions. Approximately two weeks later, single-cell clones could be transferred to 24-well plates. About 30 clones were taken as single clones from the 96-well plates, and around 120 clones were taken with cloning cylinders from the 10 cm dishes. The screening of the clones was performed by genotyping. Clones showing the desired deletion in both alleles were amplified and then stored in liquid nitrogen. Cell pellets of untreated and doxorubicin-treated p53 KO1 and p53 KO2 cells were taken for further validation by Western Blot (Supplementary Figure S1A,B). TMEM206 KO1 and TMEM206 KO2 were validated by the patch-lamp technique (Supplementary Figure S1C,D) and qPCR (Supplementary Figure S1E). p53/TMEM206 DKO1 and p53/TMEM206 DKO2 were validated by the patch-clamp technique (Supplementary Figure S1F,G) and qPCR (Supplementary Figure S1H,I). Two clones were used for functional experiments.

### 2.4. Quantitative Real-Time PCR

Total RNA was isolated from HCT116 cells using Rneasy Mini Kit (Qiagen, Hilden, Germany) following the manufacturer’s instructions. RNA concentrations were measured using a NanoPhotometer NP80 (Implen, Munich, Germany), and 2 µg RNA was used with the High-Capacity cDNA Reverse Transcription Kit (Thermo Fisher Scientific). After 1:4 dilution, cDNA was used for qPCR with the TaqMan Gene Expression Assay (Thermo Fisher Scientific). The following PCR conditions in the Bio-Rad CFX96 (BIO-RAD, Hercules, CA, USA) were used: 2 min activation at 50 °C, then 10 min at 95 °C, followed by 40 cycles of 15 s denaturation at 95 °C and 1 min annealing at 60 °C. The data were analyzed with CFX Maestro software (v1.3.1., BIO-RAD), and the analysis of each target was carried out using the comparative Ct method. TMEM206 (Taqman assay, Hs01558459_m1, Thermo Fisher Scientific) expression levels were normalized to TATA box-binding protein (TBP; Hs00427621_m1, Thermo Fisher Scientific) and RNA polymerase II subunit A (PolR2A; Hs00172187_m1, Thermo Fisher Scientific) protein expression.

### 2.5. Electrophysiology

All experiments were performed in whole-cell configuration at room temperature. Patch pipettes were pulled from borosilicate glass and had resistances between 1.5 and 3 mΩ. Voltage ramps spanning from −150 mV to +150 mV with a duration of 50 ms were applied every 2 s. The holding potential was 0 mV. Currents were acquired with an EPC-10 amplifier (HEKA, Reutlingen, Germany), recorded, and digitized with Patchmaster (v2x53, HEKA). Voltages were corrected for a liquid junction potential of 10 mV. Currents were filtered at 1 kHz and then sampled at 3 kHz. To evaluate current development, currents were extracted at −130 mV and +130 mV, normalized to the cell capacitance, and plotted versus time. To further analyze the data, IgorPro 6.37 (Wavemetrics, Lake Oswego, OR, USA) and GraphPad Prism 10.2.3 were used. Bath solutions contained 140 mM NaCl, 3 mM MgCl_2_, 0.5 mM CaCl_2_, and 10 mM HEPES, and pH was adjusted to 7.2 with HCl. In the pH 4.5 bath solution, 10 mM HEPES was replaced by 5 mM Na3-citrate, and pH was adjusted to 4.5 with citric acid. The pipette solution contained 120 mM Cs-glutamate, 10 mM HEPES, 3 mM MgCl_2_, and 20 mM Cs-BAPTA, and pH was adjusted to 7.2 with CsOH. If necessary, osmolarity was adjusted to ~310 mOsm with glucose.

### 2.6. Acid-Induced Cell Death Assay

The assessment of acid-induced cell death in HCT116 WT and p53−/−, TMEM206−/−, and double KO cells was conducted through a double staining method involving Hoechst 33342 and propidium iodide (PI) (Live-Dead Cell Viability Assay Kit; Sigma-Aldrich, St. Louis, MO, USA). WT and KO cells were incubated with DMEM medium (Thermo Fisher Scientific) of either pH 7.2 or pH 4.5 for 2.5 h at 37 °C. Both media were prepared from powdered DMEM medium. After this incubation period, the experimental solutions were replaced with a solution mixture of McCoy’s 5A medium and PBS (in a 1:1 ratio) containing Hoechst 33342 and PI. The cells were subsequently incubated for 30 min at 37 °C. For quantification, multiple 10× microscopic field images were randomly taken in an ECHO revolve 4M. The percentage of PI-positive cells within the total cell population, identified through Hoechst 33342, and the percentage of dead cells (PI-positive) within the total cell population was determined in ImageJ(1.54d) using intensity thresholds.

### 2.7. Proliferation Assay

To determine proliferation, metabolic activity assays were performed using the RealTime-Glo™ MT Cell Viability Assay (Promega, Madison, WI, USA). First, 100 μL of two-times concentrated reagent was added to the wells of a white, opaque 96-well plate. Then, 2500 cells in 100 μL were added to the reagent to reach a final reagent concentration of 1X. Luminescence signals were determined hourly for 72 h in a Spark multimode plate reader (TECAN, Männedorf, Switzerland) in a humidity chamber at 37 °C and 5% CO_2_. Luminescence signals were normalized to the corresponding measured signal intensity at t = 1 h.

### 2.8. Migration Assay

Migration assays were performed with FluoroBloks (Corning, Corning, NY, USA) in a 24-well plate format. In the bottom plate, 900 µL medium containing 10% FBS was used as an attractant, and 100,000 cells in 400 µL medium (without FBS) were seeded in the upper chamber (FluoroBloks). The 24-well plates (Corning) were incubated for 48 h at 37 °C and 5% CO_2_. Afterward, FluoroBloks with migrated cells were washed in 1 mL PBS (Thermo Fisher Scientific), then fixated in 1 mL cold methanol (Sigma-Aldrich) at -20 °C for at least 10 min. Then, they were washed 3 times with PBS for ca. 3 min, stained with 1 mL DAPI (Roche, Basel, Switzerland) (1 µg/mL) for 7 min, and then washed 3 times in 1 mL PBS for 3 min. The stained cells were kept at 4 °C before pictures were taken. Four pictures per condition were taken at 4× magnification in an ECHO revolve microscope. DAPI-positive cells were counted with ImageJ. All particles with a size less than 100 were excluded.

### 2.9. Luciferase Assay

Approximately 10,000 cells were seeded per well in a 96-well plate and incubated overnight at 37 °C and 5% CO_2_. A standard protocol for 96-well plates (120 ng DNA + 12.5 µL jetOPTIMUS buffer + 0.15 µL jetOPTIMUS reagent, both from Polyplus, Illkirch, France) was used to transfect the cells with plasmids: 40 ng pMCS-Cypridina Luc containing the TMEM206 promoter region (Supplementary Figure S2), 40 ng pCMV-red firefly Luc as an internal standard (Pierce™ Cypridina-Firefly Luciferase Dual Assay Kit; Thermo Fisher Scientific), and 40 ng of an empty vector control or a vector expressing either p53 WT or p53 MUT. Cells were incubated for 24 to 48 h at 37 °C and 5% CO_2_. After incubation, the medium was aspirated, and the cells were rinsed with 100 µL/well of 1X DPBS buffer (Thermo Fisher Scientific). After aspiration of DPBS, 100 µL per well Cell Lysis Buffer was added. The plate was shaken at moderate speed for 15 min, and complete cell lysis was checked using a light microscope. Then, 15 µL of cell lysate was added to a black flat-bottomed 96-well plate with 50 µL per well of Working Solution. Immediately, luciferase activity was detected in a Spark multimode plate reader (TECAN, Männedorf, Switzerland) using a 640 nm LP filter to capture the red firefly luciferase signal and a 480 ± 20 nm BP filter to capture the Cypridina luciferase signal.

### 2.10. TMEM206 Expression in Human Primary Colorectal Cancer Samples

The publicly available dataset GSE106582 [1] (Andrieux et al., 2018) was analyzed in the GEO2R platform (GEO2R-GEO-NCBI). TMEM206 mRNA expression was compared in 77 tumor samples versus 117 adjacent mucosa samples. Log transformation of the data was auto-detected by the GEO2R platform. Values for TMEM206 mRNA expression were exported from GEO2R to GraphPad Prism 10.2.3. To determine statistical significance, a two-tailed Mann–Whitney test was used.

### 2.11. Western Blot

Cells were harvested and resuspended in Lämmli buffer (Fischer Scientific) containing DTT (Merck, Darmstadt, Germany). Glass beads (0.5 mm diameter, Merck) were added, and lysis was performed as follows: 10 min at 65 °C with shaking at 700 rpm, 25 min vortexing, and then 10 min denaturation at 65 °C with shaking at 700 rpm. The samples were then clarified by centrifugation at 15,000 × *g* for 1 min. Equal concentrations of protein extract were loaded onto 12% gels (BioRad, Cressier, Switzerland) and transferred to PVDF membranes (Merck). The membranes were blocked for 30 min at room temperature with 5% BSA (Fisher Scientific, Hampton, NH, USA) in TBS buffer (Fisher Scientific). Afterward, the membranes were incubated while shaking with primary antibody overnight at 4 °C. The following antibodies and dilutions were used: rabbit anti-p53 antibody (Dilution 1:800, Clone 7F5, Cell Signaling Technology, Leiden, Netherlands) and mouse anti-GAPDH antibody (Dilution 1:1500, Clone FF26A, Abcam, Cambridge, UK). The membranes were washed with TBS and TBS + Tween20 (0.05 %) (Thermo Fisher) and incubated for 1.5 h at room temperature with the following secondary antibodies: goat anti-rabbit 800 (Thermo Fisher) and donkey anti-mouse 680 (LI-COR, Bad Homburg, Germany). The membranes were imaged on an Odyssey CLx Imaging System (LI-COR), and analysis was performed in Image Studio Lite version 5.2 (LI-COR).

### 2.12. Statistical Analysis

Non-normalized data sets have been tested for normal distribution by GraphPad Prism 10.2.3 normality and lognormality tests. If the non-normalized data set showed normal distribution, standard ANOVA was used for statistical analysis. If Shapiro–Wilk tests for normality failed, a Kruskal–Wallis test with Dunn’s multiple comparison test was performed. For normalized datasets, no statistical analysis was performed.

## 3. Results

### 3.1. TMEM206 mRNA Expression Is Increased in Human Primary Colorectal Cancer and p53 Knockout Increases TMEM206-Mediated Currents

**Figure 1:**
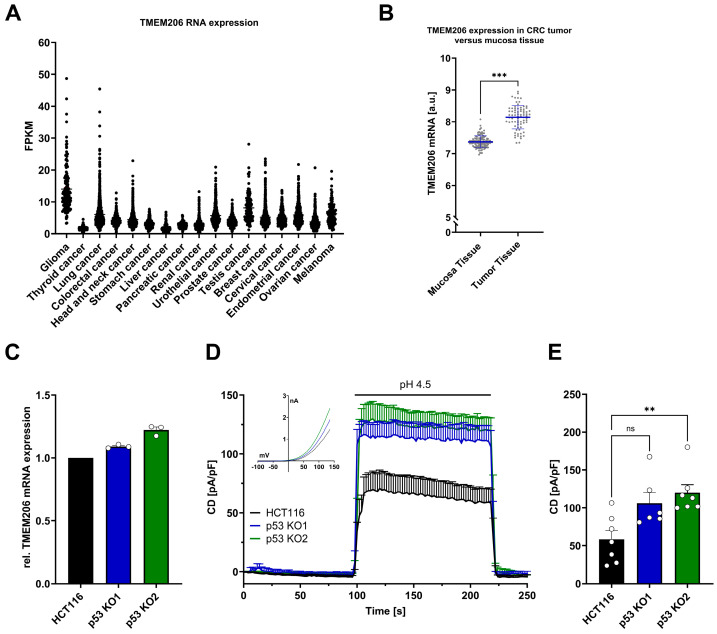
TMEM206 expression in different types of cancer, human primary CRC, TMEM206 expression, and currents in HCT116 and p53 KO cells. (**A**) TMEM206 RNA expression in different types of cancer. FPKM = number Fragments Per Kilobase of exon per Million reads. (**B**) TMEM206 expression in human primary CRC tumors versus mucosa tissue. Statistical differences were determined by a two-tailed Mann–Whitney test (n = 77 to 117 samples, mean ± SEM). (**C**) TMEM206 mRNA expression relative to TBP and Pol2RA (N = 3 independent experiments, mean ± SEM). (**D**) TMEM206-mediated current densities evoked with pH 4.5 in HCT116 cells (n = 7 cells, mean ± SEM), p53 KO1 (n = 6 cells, mean ± SEM) and p53 KO2 (n = 7 cells, mean ± SEM) and corresponding current-voltage relationships (inset). (**E**) Current densities extracted from (**D**) at t = 218 s (mean ± SEM). Statistical differences were determined by the Kruskal–Wallis test; ** *p* < 0.002, and *** *p* < 0.001.

Analysis of the TCGA dataset from Human Protein Atlas [35] revealed varying levels of TMEM206 RNA expression across different cancer types (Figure 1A). We then analyzed the publicly available dataset GSE106582 [16] with 77 samples of colorectal cancer and 117 control samples of adjacent mucosa tissues for TMEM206 mRNA expression in the GEO2R platform [36]. In adjacent colorectal mucosa tissues, TMEM206 was expressed at 7.4 arbitrary units, in contrast to 8.1 arbitrary units in colorectal cancer samples. The calculated mean fold change of 1.1 was statistically significant with *p* < 0.0001 (Figure 1B). To check if TMEM206 expression levels were affected by p53, we generated HCT116 p53 knockout (KO) cells with the CRISPR/Cas9 technique. For validation of p53 KO, we stabilized p53 with doxorubicin and evaluated p53 protein expression in HCT116 cells and both p53 KO clones (p53 KO1 and p53 KO2) by Western Blot analysis (Supplementary Figure S1A,B). In the two knockout clones, p53 KO1 and p53 KO2, TMEM206 mRNA was increased compared with HCT116 parental cells by 9% and 22 %, respectively (Figure 1C). In addition, we determined TMEM206-mediated currents activated by pH 4.5 (application as indicated, Figure 1D). The current–voltage relationships (IV) for HCT116 and both p53 knockout clones (p53 KO1 and p53 KO2) showed a profile that is characteristic of TMEM206-mediated Cl^−^ currents (inset, Figure 1D). Knockout of p53 increased current densities by 82% and 105% in p53 KO1 and p53 KO2, respectively (Figure 1E). Taken together, TMEM206 mRNA expression is increased in colorectal cancer, and knockout of p53 increases TMEM206 mRNA expression and TMEM206-mediated currents.

### 3.2. Overexpression of p53 Reduces TMEM206-Mediated Currents in HCT116 p53 KO and LNCaP Cells

**Figure 2:**
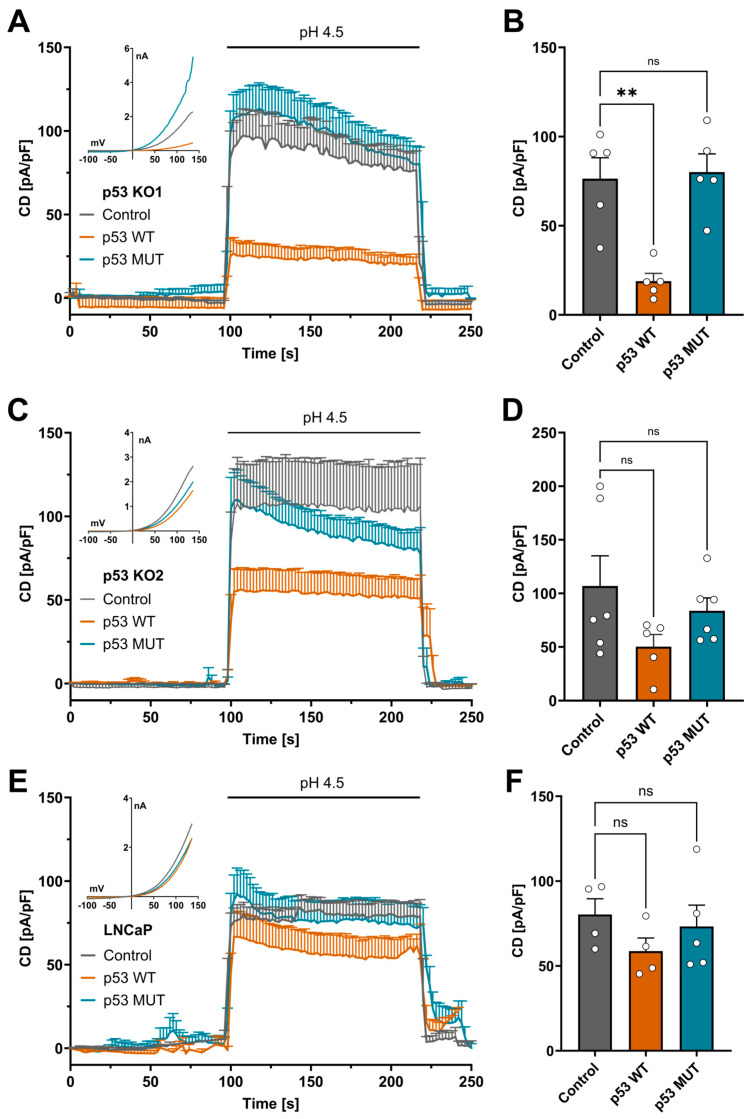
p53 rescue experiments in p53 KO and LNCaP cells. (**A**) TMEM206-mediated current densities in p53 KO1 after transient overexpression of p53 WT, p53 MUT, or the empty vector control. Currents were evoked with a pH 4.5 solution (n = 5 cells, mean ± SEM). Corresponding current–voltage relationships are shown in the inset. (**B**) Current densities extracted from (**A**) at t = 218 s (mean ± SEM). (**C**) TMEM206-mediated outward currents in p53 KO2 after transient overexpression of either p53 WT, p53 MUT, or the empty vector control. Currents were evoked with a pH 4.5 solution (n = 5 to 6 cells, mean ± SEM). Corresponding current–voltage relationships are shown in the inset. (**D**) Current densities extracted from (**C**) at t = 218 s (mean ± SEM). (**E**) TMEM206-mediated outward currents in LNCaP cells after transient overexpression of either p53 WT, p53 MUT, or the empty vector control. Currents were evoked with a pH 4.5 solution (n = 4 to 5 cells, mean ± SEM). Corresponding current–voltage relationships are shown in the inset. (**F**) Current densities extracted from (**E**) at t = 218 s (mean ± SEM). Statistical differences were determined by ordinary one-way ANOVA; ** *p* < 0.002.

To test if increased TMEM206 currents are a direct effect of p53 knockout, we transiently overexpressed wildtype p53 (p53 WT) and the non-DNA-binding mutant of p53 R175H (p53 MUT) in p53 KO1 and p53 KO2. In p53 KO1, transient overexpression of p53 WT decreased TMEM206 current density from 75.4 pA/pF to 18.9 pA/pF (Figure 2A,B). In contrast, overexpression of p53 MUT resulted in a current density of 80 pA/pF, similar to current densities in control cells (Figure 2A,B). Likewise, in p53 KO2, the current density was reduced to 50.2 pA/pF from 106.8 pA/pF after transient overexpression of p53 WT (Figure 2C,D), and the current density was only slightly reduced to 83.6 pA/pF after overexpression of p53 MUT in p53 KO2 (Figure 2C,D). To check if increased p53 expression decreases TMEM206 currents in other cancer cell lines, we transiently overexpressed p53 WT and p53 MUT in LNCaP prostate cancer cells that functionally express p53 and determined TMEM206-mediated currents. In those cells, overexpression of p53 WT decreased current density to 58.7 pA/pF compared with 80.3 pA/pF in the control transfected LNCaP cells (Figure 2E,F). Overexpression of p53 MUT only slightly reduced the current density to 73.2 pA/pF (Figure 2E,F). Taken together, transient overexpression of p53 WT reduces TMEM206 currents in both p53 KO clones and LNCaP cells that functionally express p53. Upon overexpression of p53 MUT, TMEM206 currents remain largely unchanged in p53 KO1, p53 KO2, and LNCaP cells.

### 3.3. TMEM206 Knockout Increases Acid-Induced Cell Death Dependent on p53 Expression

**Figure 3:**
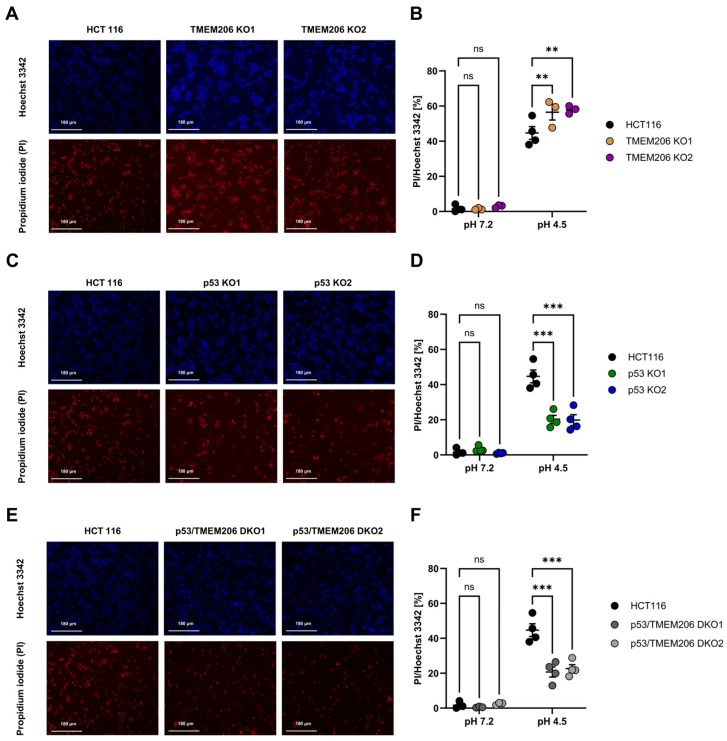
Acid-induced cell death in HCT116, p53 KO, TMEM206 KO, and p53/TMEM206 DKO. (**A**) Representative images of Hoechst 3342 (upper panel) and propidium iodide (lower panel) cell staining in HCT116 and TMEM206 KO1 and TMEM206 KO2 after 2.5 h of treatment with pH 4.5. (**B**) Statistical analysis of live-to-dead cell ratios (PI/Hoechst 3342) of HCT116 (N = 4 independent experiments, mean ± SEM) and TMEM206 KO clones (N = 3 independent experiments, mean ± SEM). (**C**) Representative images of Hoechst 3342 (upper panel) and propidium iodide (lower panel) cell staining in HCT116 (same cells as in A) and p53 KO1 and p53 KO2 after 2.5 h of treatment with pH 4.5. (**D**) Statistical analysis of live-to-dead cell ratios (PI/Hoechst 3342) of HCT116 (N = 4 independent experiments, mean ± SEM) and p53 KO clones (N = 4 independent experiments, mean ± SEM). (**E**) Representative images of Hoechst 3342 (upper panel) and propidium iodide (lower panel) cell staining in HCT116 (same cells as in A) and p53/TMEM206 DKO1 and p53/TMEM206 DKO2 after 2.5 h of treatment with pH 4.5. (**F**) Statistical analysis of live-to-dead cell ratios (PI/Hoechst 3342) of HCT116 (N = 4 independent experiments, mean ± SEM) and p53/TMEM knockout clones (N = 4 independent experiments, mean ± SEM). Statistical differences were determined by two-way ANOVA;** *p* < 0.002, *** *p* < 0.001.

TMEM206 was reported to contribute to acid-induced cell death in different cell types [6,10,13,37]. To further dissect the role of TMEM206 in acid-induced cell death, we performed an image-based cell death assay in HCT116 and TMEM206 KO clones. TMEM206 KO1 and TMEM206 KO2 were validated by the patch-clamp technique (Supplementary Figure S1C,D) and qPCR (Supplementary Figure S1E). To induce acid-induced cell death, cells were treated with a pH 4.5 solution for 2.5 h, and the ratio of live/death cells was determined with PI- and Hoechst 3342 staining and subsequent image analysis. Compared with parental cells, TMEM206 knockout cells (TMEM206 KO1 and TMEM206 KO2) were slightly but significantly more vulnerable to acid-induced cell death (Figure 3A,B). p53 KO cells (p53 KO1 and p53KO2) were significantly less sensitive to acid-induced cell death (Figure 3C,D). CRISPR/cas9-mediated knockout of TMEM206 in p53 KO cells (p53/TMEM206 DKO1 and p53/TMEM206 DKO2) was validated by the patch-clamp technique (Supplementary Figure S1F,G) and qPCR (Supplementary Figure S1H,I). Knock-out of TMEM206 in p53 KO cells did not increase acid-induced cell death compared to p53 KO cells. This non-additive effect may hint at TMEM206 as a downstream target gene of p53 (Figure 3E,F). In all control conditions, after 2.5 h of incubation with a pH 7.2 solution, we observed almost no cell death (Figure 3B,D,F).

### 3.4. TMEM206 Knockout Decreases the Cancer Hallmark Functions Including Proliferation and Migration

**Figure 4:**
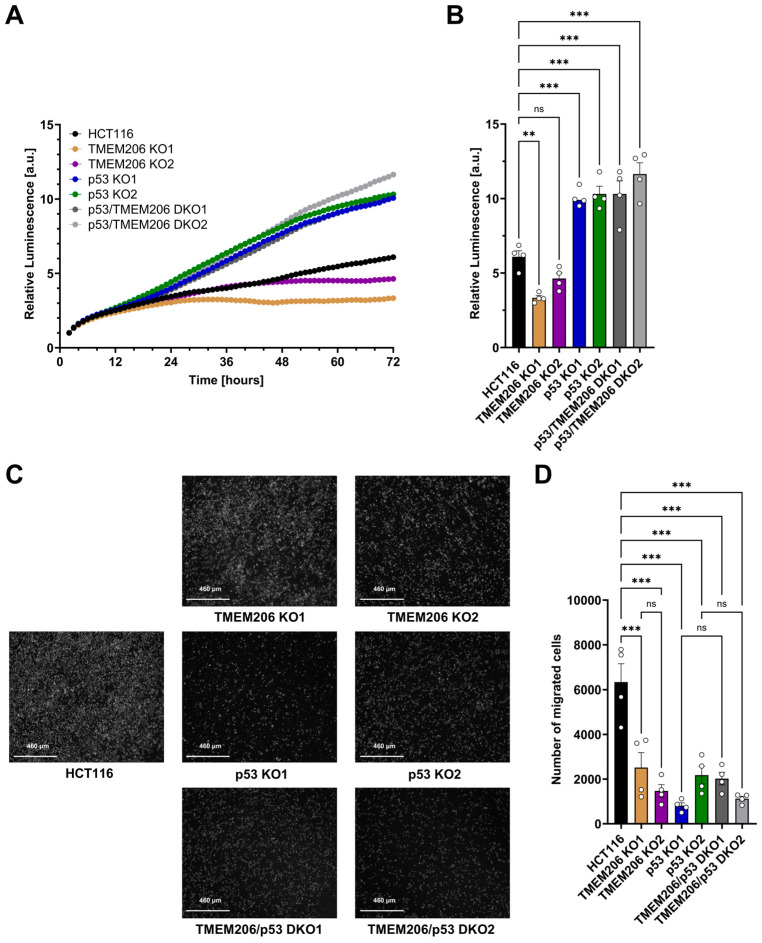
Proliferation and migration of HCT116, TMEM206 KO, p53 KO, and p53/TMEM206 DKO. (**A**) Proliferation measured over 72 h of HCT116, TMEM206 KO1 and KO2, p53 KO1 and p53 KO2, and p53/TMEM206 DKO (N = 4 independent experiments, mean). (**B**) Relative luminescence at 72 h extracted from (**A**) (mean ± SEM). (**C**) Representative images of migrated cells after 48 h (N = 4 independent experiments). (**D**) The number of migrated cells extracted from (**C**) (mean ± SEM). Statistical differences were determined by ordinary one-way ANOVA; ** *p* < 0.002, and *** *p* < 0.001.

To further investigate the role of TMEM206 and its regulator p53 on the cancer hallmark functions including proliferation and migration, we performed metabolic activity assays to measure proliferation for 72 h (Figure 4A,B). Compared with HCT116, proliferation was reduced in TMEM206 KO1 and TMEM206 KO2, suggesting a role for TMEM206 in proliferation. In contrast, proliferation was significantly increased in p53 KO1 and p53 KO2. TMEM206 KO in p53 KO cells (p53/TMEM206 DKO1 and p53/TMEM206 DKO2) did not reduce proliferation compared to p53 KO cells. This may suggest a role for TMEM206 in p53-mediated proliferation (Figure 4A,B). Cell migration was determined by trans-well migration assays (Figure 4C,D). After 48 h, compared with HCT116, migration was significantly reduced in TMEM206 KO1 and TMEM206 KO2, suggesting a role for TMEM206 in migration. In p53 KO cells, migration was reduced compared to HCT116, and further knockout of TMEM206 (p53/TMEM206 DKO1 and p53/TMEM206 DKO2) did not alter cell migration (Figure 4C,D). Taken together, here, we showed that TMEM206 contributes to proliferation and migration in CRC cells.

### 3.5. p53-Mediated Repression of TMEM206 Dependent on p21

**Figure 5:**
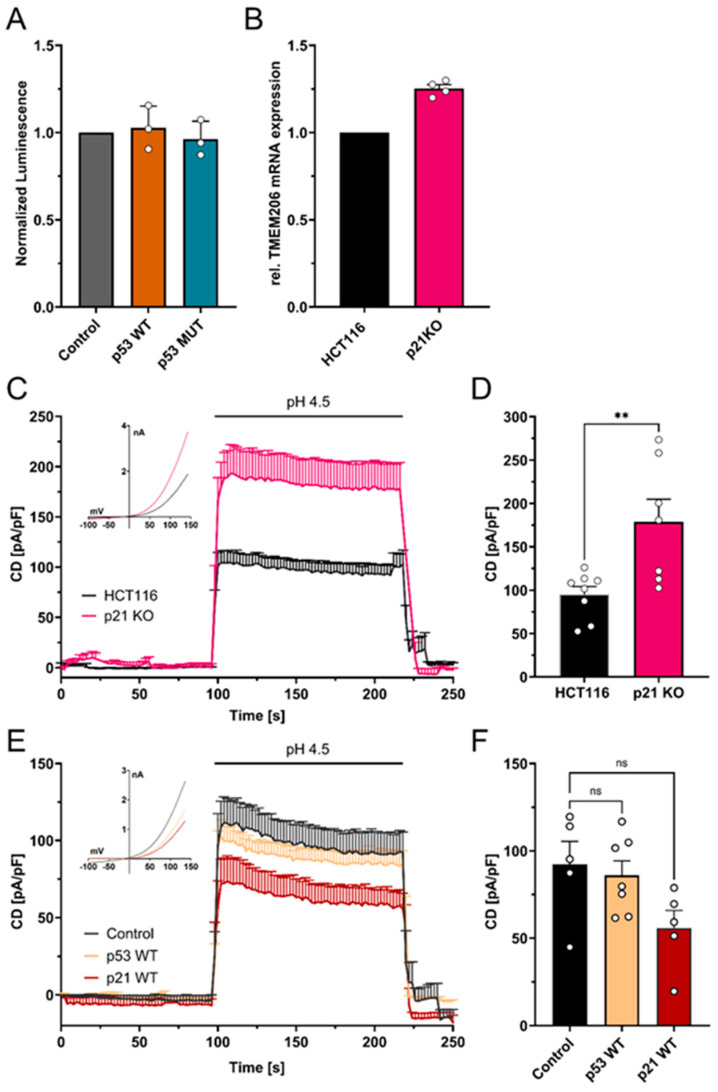
Mechanism of p53-mediated repression of TMEM206. (**A**) Normalized luminescence from dual luciferase assays after transient overexpression of p53 WT and p53 MUT in HCT116 cells (N = 3 independent experiments, mean ± SEM). (**B**) Relative mRNA expression in HCT116 and HCT116 p21 knockout cells (N = 4 independent experiments, mean ± SEM). (**C**) TMEM206-mediated outward currents in HCT116 (n = 8 cells) and HCT116 p21 knockout (n = 7 cells) cells. Currents were evoked with a pH 4.5 solution (mean ± SEM). Corresponding current–voltage relationships are shown in the inset. (**D**) Current densities extracted from (**C**) at t = 218 s (mean ± SEM). (**E**) TMEM206-mediated outward currents in HCT116 p21 KO after transient overexpression of p53 WT (n = 7 cells), p21 WT (n = 5 cells), or the empty vector control (n = 5 cells). Currents were evoked with a pH 4.5 solution (mean ± SEM). Corresponding current–voltage relationships are shown in the inset. (**F**) Current densities extracted from (**E**) at t = 218 s. Statistical differences were determined by ordinary one-way ANOVA; ** *p* < 0.002.

To test if the increase in TMEM206 mRNA and currents upon p53 knockout (Figure 1C–E) is mediated by putative p53 response elements within the TMEM206 promoter region (Supplementary Figure S2), we performed dual luciferase assays. Overexpression of p53 WT and p53 MUT did not change TMEM206 promoter activity (Figure 5A). We then tested whether TMEM206 expression is controlled by p21, a major target of p53. We determined TMEM206 mRNA expression (Figure 5B) and measured TMEM206-mediated currents in p21 deficient HCT116 cells (Figure 5C,D). Knockout of p21 has been validated earlier [38]. TMEM206 mRNA levels were increased by 25.2% upon knockout of p21 (Figure 5B). In addition, TMEM206-mediated currents increased to 178.7 pA/pF when p21 was absent compared with 94.9 pA/pF in parental cells (Figure 5C,D). Upon transient overexpression of p53 WT, TMEM206-mediated currents in HCT116 p21 KO (Figure 5E,F) remained largely unchanged. In contrast, overexpression of p21 WT reduced TMEM206-mediated currents to 60% of the currents measured in control transfected cells (Figure 5E,F). To summarize, p53 controls TMEM206 expression dependent on the p21 pathway.

## 4. Discussion

In human primary colorectal tumors (Figure 1B) and hepatocellular carcinoma, TMEM206 mRNA is upregulated and has been associated with a poor prognosis [18,19,39,40].

Here, we showed that in CRC cells, TMEM206 is regulated by p53, which is reflected by increased TMEM206 mRNA and currents in p53-deficient CRC cells. This phenotype can be rescued by transient overexpression of wildtype p53 but not by dysfunctional mutated p53. Additionally, in LNCaP prostate cancer cells that express functional p53, transient overexpression of p53 decreases TMEM206 currents, while mutated p53 fails to alter TMEM206 currents.

TMEM206 has been reported to contribute to acid-induced cell death in various cell types, where TMEM206 ablation led to reduced acid-induced cell death [10,11,12,13]. In our previous work, we used a FACS-based cell death assay to distinguish between apoptotic and necrotic cell death and did not find a clear role for TMEM206 in acid-induced cell death [17]. Although the FACS-based approach is more precise, it is prone to cell loss during a multitude of washing and staining steps. This might mask subtle differences in cell death. With the microscopy-based method used in this study, we observed a small but significant increase in acid-induced cell death when TMEM206 was knocked out. Therefore, we conclude that TMEM206 ablation decreases resistance to cell death. This is in contrast to previous studies in other cell types [6,10,13]; thus, the contribution of TMEM206 to acid-induced cell death might be cell type-dependent. Additionally, we found that acid-induced cell death is significantly decreased when p53 is absent. Additional knockout of TMEM206 in p53-deficient cells fails to increase acid-induced cell death. Thus, we did not observe an additive effect as we would expect if TMEM206 and p53 contribute independently to acid-induced cell death. This may suggest that the role of TMEM206 in acid-induced cell death is limited to p53. p53 has been demonstrated to contribute to cell death via reactive oxygen species (ROS)-dependent mechanisms [41,42]. Additionally, TMEM206 currents are dependent on intracellular ROS concentrations, as reduction in intracellular ROS decreases TMEM206-mediated currents [43,44].

Knockout of TMEM206 in CRC cells resulted in decreased proliferation, demonstrating a role for TMEM206 in proliferation, which is in line with the findings of Peng et al. for human osteosarcoma cells [20]. In contrast, knockout of p53 increased proliferation. Additional knockout of TMEM206 in p53 deficient cells did not decrease proliferation, which may hint at the hypothesis that both TMEM206 and p53 act within the same pathway. As the proliferation experiments were performed at pH 7.2, and plasma membrane TMEM206 is inactive at physiological pH and 37 °C [11,37], we can exclude a direct effect of plasma membrane TMEM206-mediated ion flux on proliferation. Peng et al. pinpointed the pro-proliferative effect of TMEM206 on the PI3K/Akt and Wnt/β-catenin pathways [20]. Both pathways are linked to p53 as dysregulated β-catenin induces p53-dependent growth arrest [45] and p53 inhibits PI3K/Akt pro-survival signaling [46]. Peng et al. demonstrated that downregulation of TMEM206 reduces migration and invasion in osteosarcoma cells [20]. In this study, we showed that the knockout of TMEM206 reduced the migration of CRC cells. Yet, the mechanism of how TMEM206 adds to cell migration remains elusive as migration and proliferation are mediated via different signaling pathways [47].

Although there is a putative p53 response element (RE) located ~2.7 kB upstream of the TMEM206 transcription start [48], overexpression of p53 did not alter TMEM206 promoter activity. Therefore, we concluded that TMEM206 repression is not mediated directly by p53-binding to the TMEM206 promoter. We found that TMEM206 is repressed by p53 via the p21 pathway. Classical targets for p21-mediated repression comprise genes with E2F or CHR (cell cycle homology region) promoter sites that are mostly found in genes important for cell cycle progression and DNA replication [49,50]. TMEM206 might be regulated by the DREAM complex [26,27,28] within the p21 pathway or other downstream targets of p21. However, we cannot draw a conclusion on the p21 downstream mechanism from our data.

## 5. Conclusions

Taken together, TMEM206 mRNA is elevated in human primary CRC tumors. TMEM206 is under the control of p53, contributes to proliferation and migration, and may reduce cell death. Dysfunctional p53 may unleash TMEM206 in CRC and add to cancer hallmark functions such as increased migration and proliferation and reduced acid-induced cell death. Mechanistically, p53 controls TMEM206 via its downstream target p21, and thus, TMEM206 may add to p53/p21-mediated oncogenic functions.

## Figures and Tables

**Table 1:** Guide RNA sequences.

Name	5′-3′	Used For
363R-fwd	CACCGCCATTGTTCAATATCGTCCG	p53 gRNA sense oligo
363R-rev	AAACCGGACGATATTGAACAATGGC	p53 gRNA antisense oligo
10R-fwd	CACCGTCGACGCTAGGATCTGACTG	p53 gRNA sense oligo
10R-rev	AAACCAGTCAGATCCTAGCGTCGAC	p53 gRNA antisense oligo
92R-fwd	CACCGCCTGAGACCGCCCCAGCCCG	TMEM206 gRNA sense oligo
92R-rev	AAACCGGGCTGGGGCGGTCTCAGGC	TMEM206 gRNA sense oligo
170F-fwd	CACCGGAGCGCTCCACATCCTACC	TMEM206 gRNA sense oligo
170F-rev	AAACGGTAGGATGTGGAGCGCTCC	TMEM206 gRNA sense oligo

## Data Availability

The original contributions presented in the study are available in the online data depository: https://doi.org/10.5281/zenodo.14018002.

## Supplementary Materials

The following supporting information can be downloaded at https://www.mdpi.com/article/10.3390/cells13221825/s1: Figure S1: Validation of HCT116 p53, TMEM206, and p53/TMEM206 knockout clones; Figure S2: TMEM206 Promoter region and adjacent sequences.
