# Supplementary material for: TMEM206 Contributes to Cancer Hallmark Functions in Colorectal Cancer Cells and Is Regulated by p53 in a p21-Dependent Manner
Source: Cells. 2024 Nov 5;13(22):1825. doi: 10.3390/cells13221825 (PMC11593115; doi:10.3390/cells13221825)
Supplement: Supplementary file 1 [file cells-13-01825-s001.zip › cells-3228895-supplementary.pdf]

Supplementary Materials for

TMEM206 Contributes to Cancer Hallmark Functions in Colorectal Cancer Cells and Is Regulated by p53 in a p21-Dependent Manner

Korollus Melek, Barbara Hauert and Sven Kappel *

Institute of Biochemistry and Molecular Medicine, University of Bern, 3012 Bern, Switzerland

* Correspondence: sven.kappel@unibe.ch

The file includes:

- **Figure S1:** Validation of HCT116 p53, TMEM206, and p53/TMEM206 knockout clones
- **Figure S2:** TMEM206 Promoter region and adjacent sequences

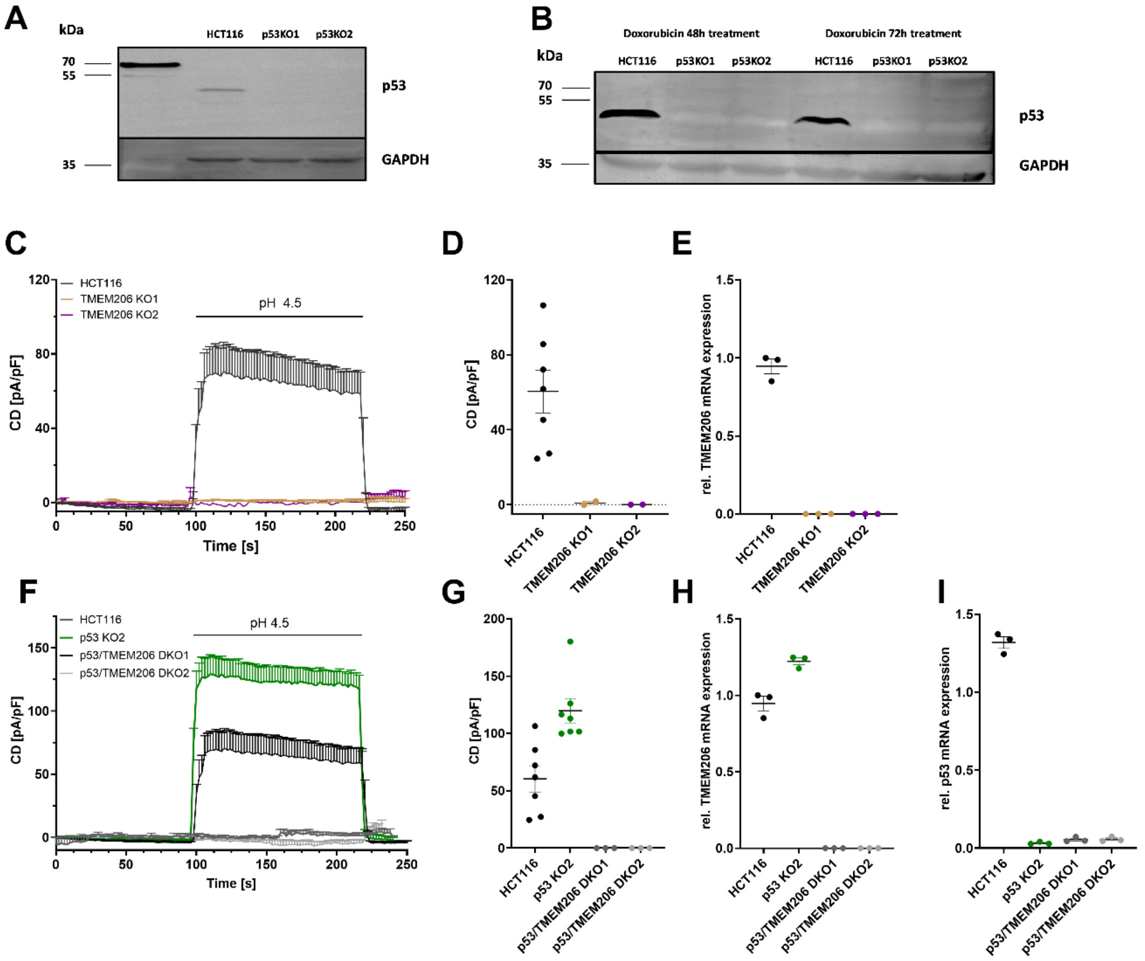

Figure S1: Validation of HCT116 p53, TMEM206, and p53/TMEM206 knockout clones. (A) p53 protein expression in HCT116 versus p53 KO1 and p53 KO2. (B) p53 protein expression in HCT116 versus p53 KO1 and p53 KO2 48 and 72 hours after p53-stabilizing doxorubicin treatment. (C) TMEM206-mediated currents in HCT116 versus TMEM206 KO1 and TMEM206 KO2 as functional readout of protein expression. (D) Current densities extracted from (C) at t = 218 s. (E) Relative TMEM206 mRNA expression in HCT116 versus TMEM206 KO1 and TMEM206 KO2. (F) TMEM206-mediated currents in p53 KO2 versus p53/TMEM206 DKO1 and versus p53/TMEM206 DKO2 as functional readout of TMEM206 protein expression. Currents of HCT116 have been included for reference. (G) Current densities extracted from (F) at t = 218 s. (H) Relative TMEM206 mRNA expression in p53 KO2 versus p53/TMEM206 DKO1 and p53/TMEM206 DKO2. (I) Relative p53 mRNA expression in p53 KO2 versus p53/TMEM206 DKO1 and p53/TMEM206 DKO2.

```
           0987654321098765432109876543210987654321098765432109876543210987654321098765432109876543210987654321
212417663  TAAAAAAAACCAAAACAACAACAACAAACCTAGCTTTGGGAAAGTCCGCCCTGTAGAGCTGAGATGAGGAGAAGTCAGCAGAGAGGCTGA
212417573  GAAAGAGCAGTAACTGTAACTGTAATAGGCCTGTTGCCTGATGTGTGCAGCAAGTCAATATGCCAAGACGGAGTTGCAGTAGAAAAAGAG
212417483  GCTTACCAATAGGGCCTCCAATCGAGGAGATGGAGGGGAACCTCAAACCCTCTCTCAAGGAGTTTGCGGCTTTTCTAAGGGTTTTTAAGG
212417393  AGTAGGCTGAAGTGTGGAGACTGCTGATTGGCCAAAGGGAACAGGGTGAAATCATTGGACAGAGAGAGAAGCAGCTGTATTCTCATGCTG
212417303  ATTCCATTCCTCTGTTGGTGTCTTCAAACTTGTTGGTGTCAGCTGTTTCACTGGAATTTGTGATCTGAAAAACATTTTAAGCAATTTTTT
212417213  TTTTTGAGACAGAATTTTGCTATTGTCGCCCAGGCTGGAGTGCAATGGTGTGATCTTGGCTCACTGCAACCTCTGCCTCCCGGGTTCAAG
212417123  CGATTCTCCTGCCTCAGCCTCCTGAGTAGGGAGGCTAGGGAGGCTGGGATTATAGGTGCCCGCCACCATGCCTGGTTAATTTTTGTATTT
212417033  TTAGTAAAGATGAAGTTTTACCATGTTGGCCAGGCTGGTCTCGAACTCCTGACCTCAGGCAATCCGCCCGCCTCGGCCTCCTAAAGAGTT
212416943  GGGATTACAGGCGTGAGCCACTGGGCCTGGCCTTAAACAATTCTTAAGCAAAAGCCTTATGATTCCAATGTCAGAAATCCTACCTGTAGG
212416853  AGCAGTGGAGATGCAAATGGTCAGTATCTAGTGCTACATGACTTTTGGCTACAAGGAAGTGGGTCAAATTACAGCCTGATTAATGCTTAA
212416763  TTATAACTCTATTTCTGTGCAGAATTCGTGTTAACCCTGTGAGGATGGCTTCAAAGTGAGGTAGGAGCAAGCCGGGAGTGTGTGGTGTCC
212416673  TGGAAGCCAGGGAGGAACGCGTATCAAGGAGGAGGGAGGATCAGCCAAGTCCACTGTGGCTGATAAATCAAGAAAGAGGAGGATGGGAAA
212416583  ATGGCCATCTGATGTGACAACACTGAGGTCACTTGTGACCTTGACAAGCACTGTTCCAGTGGAGTGGAGGGGAGTAAAGCCTGACTGGTG
212416493  TGTGCTCATGAAGGAATGGGAGAGAGGACTTGGGGGCTGCAAGGACAGAACATTATTTACATGTTTATGATCTGTCTCTCCACAGTTGCA
212416403  TGAAAGAGCCACGAAAGCCAGGACCCAAGGGTATCTTATCCATTTCTGTATCCCAGTGCCCAGCACATTGTATTTGTTAAGCCATATTTT
212416313  ATATTTTATTAGCCAGTATCTCCTTCAAATGTGGGAGCCCATAATTTTCCAGTTTGGTCACACCAACAAAGACTAGAGAGGAATGACAGG
212416223  GTTGGAATAGAATTCTCTGGTTCCTTCCAGCTCTAATATTCTATGATGGTGTGAACTTGTTCTATCAAGTACCATTGTATTAACACAGTA
212416133  AGGAATTTAATTAGTGGTCTGCTATTGTTAATTTGTTTTGGTAAGCAACTGTTAAATGAAATGGCTGGAATCAGTGTTCCATTCATAAAT
212416043  TTAACAAACATTTGTCGAGTGCCTACAATGTGTTAGGCACTATATATATTGTCCCATATAACAGATTTAAGAGCAGAGGGCCTAGGATAG
212415953  GGGTGAGTGGTCAGGAGAGGTGATGGGGACCTCCCATTGCTAATGCTCCAGGAGGTCCAGGGTTCTTTGATGTGGCTGGCACTATTCTGC
212415863  TTATCACTGAGGAGAGAAGAGGCTCCCAGTGGCCTGAGTGGAGGGGTTACCAAGCTGCAGCTGCAAGGGACACAGAGCAGCATTCACCAA
212415773  GGCACAACAAGCAGCCTCTATCTGCTTCACCACTGAGGAGCCGAGGGCCCTCACACAACAGCTGTTTCAGGCAGTGGGAGGAGAGGAAGA
212415683  TGTGGTCTTCAGGTCCACCCTGCCACAGGGCCAACACCAGATTTGACGCCACATCTAGCGCTTGAGGCTTCTTCTGTGGCTGAACAGCGG
212415593  CTTTAGGCTTTCTCTGCACGATCCTTCCTTTCTGCTTTCATAGGCCTTGTCAGGCAGGGCAGGGTTTAGCAGTTGCATTCTAAGGCGAGG
212415503  AGAGGAGGGCTCAGAGCATTGCAAGACCTAAAGGGGAGGGTCAGAGCCAGGACAAGAACCTAGGGGTACTCCTGCCCCTGGAATAATGGA
212415413  GAGAACATGGGCTCCGCAGTCTACCCCTGGATGAAATAATCTTCATTTGATTACCGCTCCACCCGCATCCTTTCTGAAACAAAGTACCTC
212415323  AGTTTCCTCATTTGTAAATTAGGGAGAAAAATGTCCTTCTTACAGGGTTCCTTGCAGAATCAACGGCATAATGTGAGTGAGAGCGCTTTG
212415233  TAACTAACATACAAAGTGCTCTCCAGACATTTATTACCGTTTTAAACCGCCAGGCCAAGCTTCCTCTTGCAATTATAGATTAGGGGCTCC
212415143  TACTCATCTGACTTTGTTCCACTTTCCACTCCTCTTCTCCGTCCCTCCCCCTCCAACAAAGAGAGGAAGTGAGAGACGCAGAGGCACGCG
212415053  TCTAAGGACCAGCTTAGCTGGCCCAGGGTCACATGCAGGCCTCCGCCACAGGGGTGAGAGCTGGTGTGAGGGGCGCCCCCGCCCAGAACT
212414963  ACATTTCCCATCAACACTTTCGCGACTCCTGGGCGGCAGCGGCCAGGCGCATGCGCCGAGTATCGCCACGCCTGGTCTCTGGGACGCCCC
212414873  TCCGGACCGGTTTCGCCTCGCGGAGCCGGTAGGTCCAGGTGCAGCGGCCGCAGTGCTGCGTCCGTGCGCCGCGGGCTGGGGCGGTCTCAG
212414783  GTGTGCCGAAGCTCTGGTCAGTGCCATGATCCGGCAGGAGCGCTCCACATCCTACCAGGAGGTACGAGGTTGTGAGAGACGGAAGGGAAG
212414693  TTTGAGGCGGAGGAGCAGGGTCCCGGGGGCCTGGGCGGATGCGGGGCGGGGGGTGTCGGGGCTGCGCGGCAGAGGGCTCTTCCGAGGGAC
212414603  CGACGGGTGCGTCAGGGGAGGGCTCCCGGGGTGCAGAGGAGCGGCCCAGGGGCAGGAAGGAAGCGGGGCAGCGGAGCGGGCGCCCAGCGT
212414513  TCTCCGTATGCTGCGGGTTGCATGGCCGAGGTCCGGAGTAGGGTCCCCGGCGCCGCGACCCGTAACCAGCAGGTGAATGATTGAAGCTAC
212414423  CTGAGGCCCAGCCTTCCGCGCTCCCCGCCGGAGCGGCAGCCCAATGGCCCAGAGGAACCCGGCGTTCTTTGCAACAAGTGGAGCGCCCGC
212414333  GCCCTCCTCCCGGCGAGATCTGATGCGCGGTCGGCATCGTCGGCATCCTCCCCCTCCAAGCCCAAGGCCTCGCTGTCCCCGCGGCTGGTG
212414243  ACAGGATTGATTACCTGTTTTTTGGTGACCACCTAACTTTGAAAAAGTGCCTTCATGGACTCTGCTAGTTCTCGCTCCTCTTCGTCTCTC
212414153  CCTCCAGTCTCACCTCCTCCCACGACTTTATCACTTTCCAGTTCTTTGTCTCTTCTCTGTCCTGCGCTGCAGGCTGTGCGTCCAGCGCTT
212414063  CCTTCAAGCAAGCCCTGCCTCCTCACTTCTCCAGCTGTATTAGTGAAACTGCTCTCCTCCTCCGCCTCCACTTCTCGGCCCCCAAACCTT
212413973  GGTCATCTTTGGCAACCGTCCTCTTCTGTGCCCCTCCATCGTGCCGCCTCATTGGCCAAAGTCAGGCAGTTCCAGGTCGGTCCTCCTCCA
212413883  GTTTGTGTCCCTGTGTTTGCTGTAACCTCTGAGTGCGATACTGTGTTACCATCGCTCCCTTTAGTCTCTTATACTCTCCAGATTTGCCTT
212413793  GAGGCCCTGCCTTCACCTCGGAGCATTCCTTTTTTCTCAGACCTGACGCTGCCCTTCGTCTGCCCAGAACAGTCCAGACTTCTCAGCAGG
```

CCTGGGCGGCAGCGGCCAGGCGCATGCGCCGAGTATCGCCACGCCTGGTCTCTGGGACGC   Promoter region

AACCTAGCTTTGGGAAAGTCC   p53 Response element

CTCTGGGACGCCCCTCCGGACCGGTTTCGCCTCGCGGAGCCGGTAGGTCCAGGTGCAGCGGCCGCAGTGCTGCGTCCGTGCGCCGCGGGCTGGG
GCGGTCTCAG
GTGTGCCGAAGCTCTGGTCAGTGCCATGATCCGGCAGGAGCGCTCCACATCCTACCAGGAG   Exon 1

ATG   START CODON

GTACGAGGTTGTGAGAGACGGAAGG   START INTRON 1

Figure S2: TMEM206 promoter region and adjacent sequences. Putative p53 response element highlighted in light blue.
